# Changes in the Molecular Forces and Rheological Properties of Pork Myosin with Sodium Bicarbonate

**DOI:** 10.3390/foods15193539

**Published:** 2026-10-04

**Authors:** Yu Zheng, Zhan-Peng Zhu, Qin Hou, Wang-Li Cheng, Xue-Hua Zhang, Zhuang-Li Kang

**Affiliations:** 1 Key Laboratory of Chinese Cuisine Intangible Cultural Heritage Technology Inheritance, School of Tourism and Cuisine, Yangzhou University, Yangzhou 225127, China; 2 School of Food Science, Henan Institute of Science and Technology, Xinxiang 453003, China

**Keywords:** turbidity, rheological property, surface hydrophobicity, apparent viscosity, whiteness

## Abstract

To investigate how sodium bicarbonate concentrations (0, 10, 20, 30, 40, and 50 mM) modulate the turbidity and molecular interactions of pork myosin, the pH, turbidity, particle size, active and total thiol content, surface hydrophobicity, rheology properties, and chemical forces were analyzed. Results revealed that increasing sodium bicarbonate concentration from 0 to 50 mM significantly elevated (*p* < 0.05) pH, surface hydrophobicity, apparent viscosity, active thiol content, and storage modulus (G′), while significantly reducing (*p* < 0.05) turbidity, mean particle size, gel whiteness, and ionic bonding strength. The alkalinization-induced pH reduced protein turbidity and enhanced molecular unfolding, thereby exposing hydrophobic regions and reactive thiol groups; this promoted hydrophobic interactions and facilitated disulfide bond formation during heat-induced gelation. Consequently, higher sodium bicarbonate levels (40 and 50 mM) markedly strengthened intermolecular forces and improved the rheological performance of the resulting myosin gels.

## 1. Introduction

Myosin, the predominant component of myofibrillar protein, comprising 50 to 55% of its content, exhibits excellent heat-induced gel capabilities. Hence, improving the functional characteristics of myosin is crucial for enhancing the quality of meat products [1]. Myosin undergoes self-assembly in solution, influenced by factors such as protein concentration, pH, temperature, and ionic strength. Through intermolecular interactions and binding, stable polymers are formed [1]. Some researchers discovered that adjustments to pH and ionic strength can alter the protein structure and conformation of myosin, with pH having a more pronounced effect on myosin structure and conformation [2]. For example, enhancing the pH and ionic strength accelerate the denaturation of the myosin structure, enhance the surface hydrophobicity, increase the β-sheet content, and reduce the Ca^2+^-ATPase activity [3,4].

Sodium bicarbonate is frequently utilized as a phosphate substitute in meat products. It enhances the juiciness and palatability of meat, reduces cooking losses and shear force, and thereby improving overall eating quality [5,6]. Furthermore, sodium bicarbonate disrupts the connective tissue in muscle, leading to looser muscle structure and enhanced product tenderness. Additionally, its acid anion alters the muscle’s pH, causing myofibrillar proteins to deviate from their isoelectric point, which in turn boosts protein solubility and gel properties [7]. Chantarasuwan, Benjakul, and Visessanguan [8] revealed that the incorporation of sodium bicarbonate and sodium chloride into Pacific white shrimp resulted in increased solubility and sulfhydryl content of actomyosin, decreased ATPase activity, enhanced hydrophobic interactions, induced myosin head denaturation, and led to actomyosin dissociation and consequently the loss of its fibrous structure. Saleem, Hasnain, and Ahmad [9] discovered that the addition of high concentrations of sodium bicarbonate could alter protein substructures, including the dissociation of myosin light chain and regulatory proteins of the actin complex. Our previous research has shown that compared with sodium chloride, sodium bicarbonate can increase the solubility of pig myofibrillar protein, reduce the particle size of protein aggregation, and induce the formation of more β-sheet structures [10]. Although prior studies have documented the modulatory effects of sodium bicarbonate on pork myofibrillar proteins, few investigations have specifically addressed its impact on myosin. Therefore, this study established a solution system comprising sodium bicarbonate and pork myosin to investigate the mechanism through which varying amounts of sodium bicarbonate affect the molecular forces and rheological properties of pork myosin.

## 2. Materials and Methods

### 2.1. Raw Materials

Chilled *longissimus lumborum* muscle (12–24 h after slaughter, pH 5.66 ± 0.02; protein 20.19 ± 0.63% *w*/*w*) was obtained from 12 healthy pigs, sourced in three independent batches (4 pigs per batch) from a local market (Xinxiang, China). Muscles were trimmed to remove visible connective tissue and adipose tissue, then coarsely ground using a laboratory-scale grinder equipped with a 6 mm die plate (model JR-120, Zhucheng Haicheng Machinery Co., Ltd., Zhucheng, China). Ground samples from each batch were homogenized thoroughly to ensure compositional uniformity. All reagents were of analytical grade.

### 2.2. Extraction of Pork Myosin

Myosin was isolated according to the method described by Liu et al. [11], with all procedures performed under strictly controlled under 4 °C. Briefly, 100 g of ground pork was taken and mixed with five times its volume of solution A (0.1 M Tris-HCl, 20 mM EDTA). The suspension was homogenized using an Ultra-Turrax T25 disperser (IKA, Staufen, Germany) for 2 min at 12,000 rpm on ice, followed by centrifugation at 3000× *g* for 10 min at 4 °C using a centrifuge (Sorvall LYNX4000, Thermo Fisher Scientific, Langenselbold, Germany). The precipitate was collected and dissolved in three times its volume of “Hasselbach–Schneider” (H-S) solution (0.4 M KCl, 0.1 M KH_2_PO_4_/K_2_HPO_4_, 10 mM sodium pyrophosphate, 1 mM MgCl_2_, 2 mM EGTA, pH 6.4). Homogenization was repeated, and the mixture was centrifuged at 8000× *g* for 15 min at 4 °C. The suspension was collected and diluted with 9 times its volume of pre-cooled ultrapure water, allowing it to settle overnight. The suspension was then siphoned off, and the remaining solution was centrifuged at 12,000× *g* for 12 min at 4 °C. The precipitate was collected and mixed with three times its volume of 0.1 M KCl (pH 7.0). Stirring was performed, followed by centrifugation at 8000× *g* for 12 min at 4 °C. The resulting precipitate was myosin. The extracted myosin was stored in a refrigerator at 4 °C within 24 h. The myosin content was determined using the biuret method, with bovine serum albumin serving as the standard protein.

### 2.3. Preparation of Pork Myosin Solution and Gel

Pork myosin was dissolved in 1.0 mM KCl buffer (pH 7.0) to a final concentration of 20 mg/mL. Sodium bicarbonate was added at six concentrations (0, 10, 20, 30, 40, and 50 mM) to establish the experimental myosin solution system. Three independent replicates were performed using chilled *longissimus lumborum* muscle from different animals. For each replicate, twenty 4 mL aliquots of myosin solution (one per concentration × three replicates × 20 total per replicate) were prepared in 5 mL beakers and sealed with plastic film. Of these, eight samples per concentration were heated in a water bath at 80 °C for 15 min to induce gelation; the remaining twelve samples per concentration were stored at 4 °C and analyzed within 24 h.

### 2.4. pH Determination

Pork myosin was diluted to 5 mg/mL. The pH of each treatment was then determined by a portable pH meter (PHSJ-4F, Shanghai Leimei Instrument Co., Ltd., Shanghai, China) with the automatic temperature compensation mode.

### 2.5. Turbidity Determination

Pork myosin was diluted to 2 mg/mL, and the absorbance of each myosin sample was determined at a wavelength of 340 nm using an ultraviolet spectrophotometer (722N, Shanghai Qinghua Technology Instrument Co., Ltd., Shanghai, China).

### 2.6. Particle Size Determination

Pork myosin was diluted to 0.1 mg/mL. Particle size was measured using a nanoparticle analyzer (Zetasizer v7.11,Malvern Instruments Ltd., Malvern, England), and the value of Z-ave was obtained.

### 2.7. Determination of Active and Total Sulfhydryl Contents

Active and total sulfhydryl contents of pork myosin were determined according to Kang et al. [12]. Briefly, 1.5 mL of myosin solution (5 mg/mL) was mixed with 10.0 mL of Tris–glycine buffer (0.086 mol/L Tris, 0.09 mol/L glycine, 4 mmol/L EDTA, pH 8.0). Then, 50 μL of Ellman’s reagent (prepared by dissolving 4 mg 5,5′-dithiobis-(2-nitrobenzoic acid) (DTNB) in 1 mL Tris–glycine buffer) was added, and the mixture was vortexed for 30 s. After incubation at 25 ± 1 °C for 60 min in the dark, the reaction was terminated by centrifugation at 12,000× *g* for 10 min. The absorbance of the supernatant was measured at 412 nm using a UV–vis spectrophotometer. Active sulfhydryl content was calculated directly from the absorbance; total sulfhydryl content was determined similarly after prior reduction with 20 mmol/L β-mercaptoethanol for 60 min under nitrogen atmosphere.

### 2.8. Determination of Surface Hydrophobicity

Surface hydrophobicity was measured following Li et al. [10] with minor modifications. Briefly, 1 mL of myosin solution (2 mg/mL) was mixed with 0.2 mL of bromophenol blue (BPB) solution (1 mg/mL in water), vortexed for 10 min at room temperature, and then centrifuged at 2000× *g* for 15 min. The supernatant was diluted 10-fold with distilled water, and absorbance was recorded at 595 nm using a UV–vis spectrophotometer. Surface hydrophobicity (H_0_, μg BPB bound per mg protein) was calculated as:

H_0_ (μg) = 200 μg × (OD_control_ − OD_sample_)/OD_control_

where OD_control_ is the absorbance of the BPB–protein mixture before centrifugation, and OD_sample_ is the absorbance of the diluted supernatant.

### 2.9. Determination of Apparent Viscosity

Apparent viscosity was measured using a Haake Mars 60 rotational dynamic rheometer (Thermo Fisher Scientific, Bochum, Germany) equipped with a parallel-plate geometry (P35 TiL; plate diameter: 35 mm; gap: 1 mm). Prior to measurement, the raw pork myosin solution was evenly dispensed onto the lower plate, and any excess sample was carefully removed to ensure a uniform gap of 1 mm. A thin layer of silicone oil was applied to the outer rim of the plate assembly to prevent moisture loss during testing. The sample was equilibrated at 25 °C for 5 min before data acquisition. Rheological measurements were conducted under controlled conditions: shear rate range of 10 s^−1^–1000 s^−1^, constant temperature of 25 °C, and total shear duration of 330 s. Apparent viscosity values were calculated from the steady-state flow curves and fitted to the Power-law model.

### 2.10. Determination of UV Absorption Spectrum

Pork myosin was diluted to 0.4 mg/mL and measured by a visible spectrophotometer (Shanghai Yoke Instrument Co., Ltd., Shanghai, China). Parameters were as follows: spectral wavelength scanning range 190–400 nm, interval 1 nm, scanning speed 240 nm/min.

### 2.11. Determination of Rheological Properties

Rheological properties of raw pork myosin solutions were measured using a Haake Mars 60 Rotational Dynamic Rheometer (Thermo Fisher Scientific, Bochum, Germany). Lay the myosin solution evenly on the plate and wipe off the excessive sample. Frequency scanning parameter setting were as follows: probe model P35 TiL, plate diameter 35 mm, gap 1 mm, frequency scanning range 0.1 Hz–10 Hz, temperature 25 °C, strain value 1%.

### 2.12. Whiteness Determination

After refrigeration at 4 °C for 12 h, pork myosin gels were carefully removed from the beakers, sliced into uniform thin sections, and analyzed for whiteness within 90 s using a CR-400 colorimeter (Konica Minolta, Osaka, Japan) equipped with an 8 mm aperture, a 10° standard observer angle, and D65 illuminant. Prior to measurement, the instrument was calibrated against a standard white tile. Whiteness was calculated as:

(1) $$\mathrm{Whiteness}\;=\;100\;-\;\sqrt{{(100\;-\;L^{*})}^{2}\;+\;{a^{*}}^{2}\;+\;{b^{*}}^{2}}$$

### 2.13. Determination of Chemical Force

Chemical forces were quantified following Yang et al. [13] with minor modifications. Briefly, 1.0 g of myosin gel was placed into a 10 mL centrifuge tube, and 5 mL of each extraction solution was added sequentially: SA (0.05 M NaCl), SB (0.6 M NaCl), SC (0.6 M NaCl + 1.5 M urea), SD (0.6 M NaCl + 8 M urea), and SE (0.6 M NaCl + 8 M urea + 1.5% SDS). Samples were vortexed for 30 s, incubated at 4 °C for 1 h, then centrifuged at 10,000× *g* for 15 min at 4 °C. Aliquots (1 mL) of supernatant were mixed with 4 mL biuret reagent, and absorbance at 540 nm was measured spectrophotometrically. Protein concentration was determined from a bovine serum albumin (BSA) standard curve. The chemical forces were calculated as:

Ionic bond = protein content in SB − protein content in SA

Hydrogen bond = protein content in SC − protein content in SB

Hydrophobic interaction = protein content in SD − protein content in SC

Disulfide bond = protein content in SE − protein content in SD

### 2.14. Statistical Analysis

All experiments were performed in triplicate (*n* = 3). Data were analyzed by one-way analysis of variance (ANOVA) using the General Linear Model (GLM) procedure in SPSS Statistics v.26.0 (IBM Corp., Armonk, NY, USA). Sodium bicarbonate concentration was treated as a fixed factor, while experimental replicate was included as a random factor to account for batch-to-batch variability. Significant differences between means were compared by the LSD procedure. Differences were considered statistically significant at *p* < 0.05, and results were expressed as mean ± SE.

## 3. Results and Discussion

### 3.1. pH

**Figure 1:**
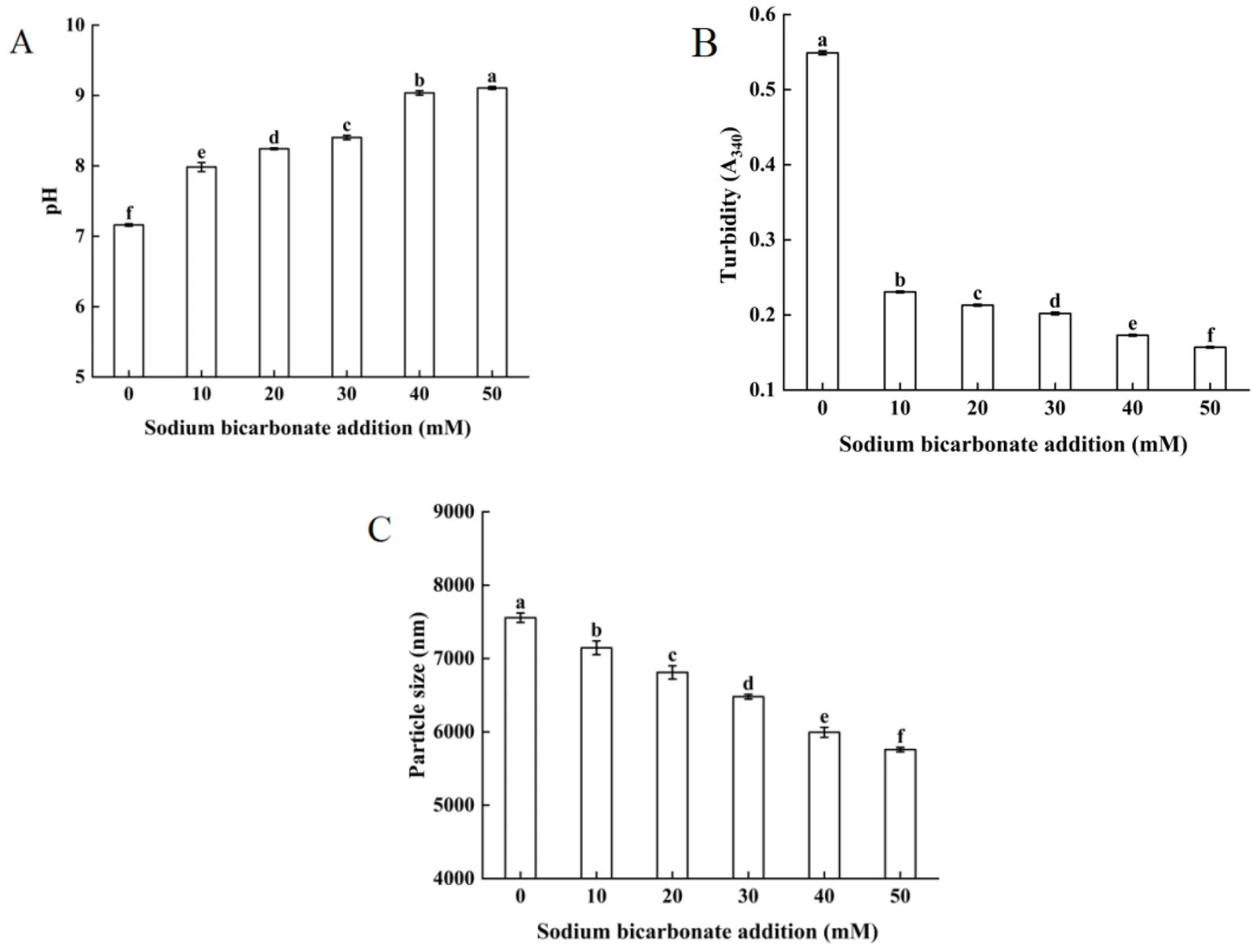
Changes in the pH (**A**), turbidity (**B**) and particle size (**C**) of pork myosin with different sodium bicarbonate concentrations. Each value represents the mean ± SE, *n* = 3. a–f Different parameter superscripts indicate significant differences (*p* < 0.05).

Figure 1A shows the effect of sodium bicarbonate concentration on the pH of pork myosin solutions. pH increased significantly with increasing sodium bicarbonate addition (*p* < 0.05), but the rate of increase attenuated beyond 30 mM. As a salt of a weak acid (carbonic acid) and strong base (sodium hydroxide), sodium bicarbonate hydrolyzes in aqueous solution to yield bicarbonate (HCO_3_^−^) and hydroxide (OH^−^) ions, thereby elevating solution alkalinity. Consequently, progressive addition of sodium bicarbonate shifts the myosin solution further from neutrality, resulting in a higher pH. Consistent with this, Li et al. [10] reported that substituting NaCl with sodium bicarbonate in pork myofibrillar protein solutions raised the pH from 5.68 to 6.27. Similarly, Lu et al. [2] observed a 0.13-unit pH increase in phosphorus-free chicken batter when sodium tripolyphosphate was replaced by an equimolar amount of sodium bicarbonate; moreover, pH rose significantly with increasing sodium bicarbonate dosage [14]. Zou et al. [15] also demonstrated that increasing the addition of sodium bicarbonate from 0 g/kg to 6 g/kg significantly elevated the pH by approximately 1.04 units of PSE meat myofibrillar protein.

### 3.2. Turbidity

As shown in Figure 1B, turbidity of the myosin solution decreased significantly with increasing sodium bicarbonate concentration (*p* < 0.05). This reduction reflects enhanced myosin solubility, attributable to the elevated pH shifting the protein away from its isoelectric point (pI ≈ 5.4), thereby increasing net surface charge and electrostatic repulsion between molecules. Yu et al. [16] reported that beef myosin aggregates readily at low pH near its isoelectric point, whereas deviation from the isoelectric point increases net charge and suppresses aggregation, resulting in lower turbidity. Furthermore, the degree of myosin deconstruction is influenced by strong electrostatic repulsion between proteins, hindering the formation of an orderly and compact network structure and consequently reducing turbidity [17].

### 3.3. Particle Size

Particle size serves as a critical indicator for characterizing the degree of protein aggregation. Figure 1C reveals a significant decrease in the average particle size of pork myosin solutions with increasing sodium bicarbonate concentration (*p* < 0.05). This trend correlates directly with the pH elevation (Figure 1A): as pH rises, electrostatic repulsion between myosin molecules intensifies, promoting dissociation of pre-existing aggregates and inhibiting re-aggregation. The observed reduction in particle size aligns quantitatively with the concurrent decline in turbidity (Figure 1B). Supporting this mechanism, Yang et al. [18] demonstrated that as the pH of myosin deviates further from its isoelectric point, electrostatic repulsion and low-speed shear forces between proteins accelerate the dissociation of myosin aggregates, thereby reducing the average particle size and improving solubility. Likewise, Gao et al. [19] found that the particle size of bighead carp myosin aggregates decreases with rising pH, with the extent of reduction being negatively correlated with the pH increase.

### 3.4. Total Sulfhydryl and Active Sulfhydryl Groups

**Figure 2:**
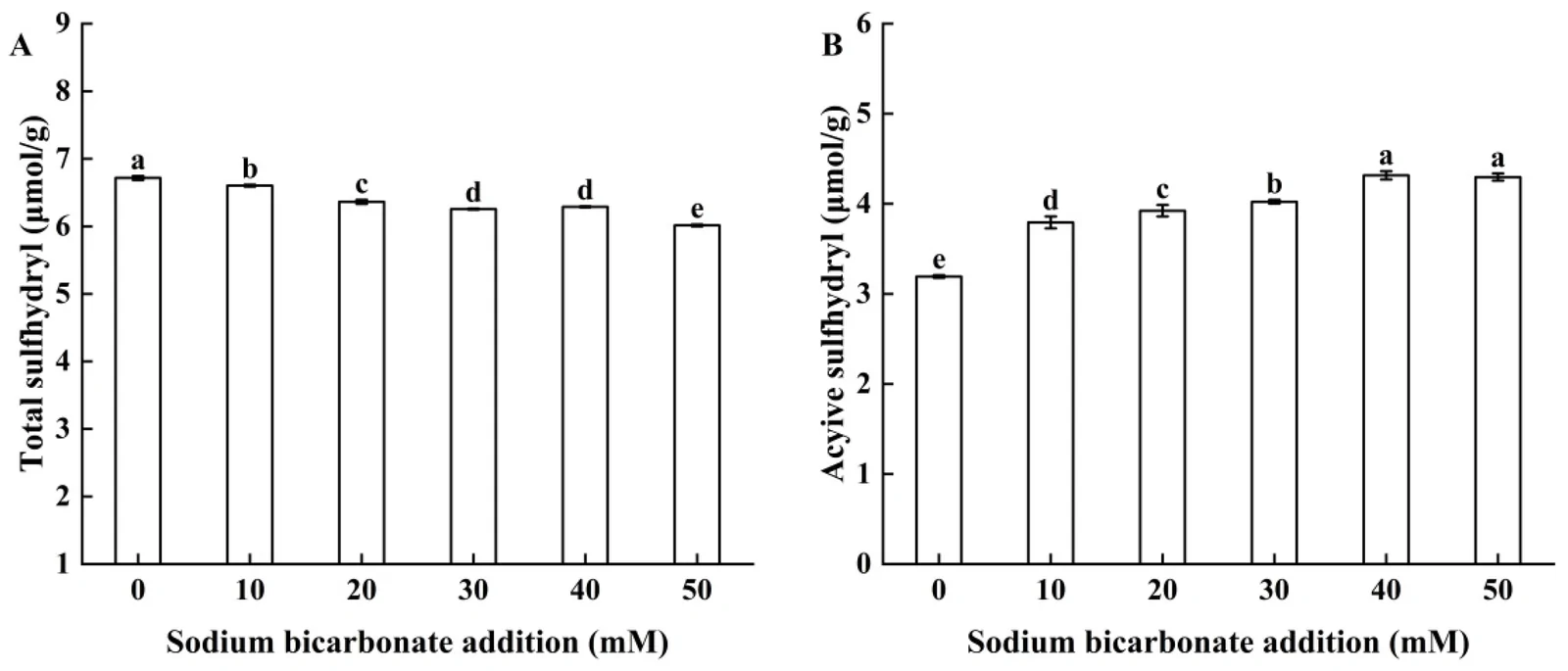
Changes in the total sulfhydryl groups (**A**) and active sulfhydryl groups (**B**) of pork myosin with different sodium bicarbonate concentrations. Each value represents the mean ± SE, *n* = 3. (a–e): Different parameter superscripts indicate significant differences (*p* < 0.05).

Sulfhydryl groups are critical determinants of protein structural stability, and changes in total sulfhydryl content serve as a well-established indicator of protein denaturation. Specifically, alterations in myosin sulfhydryl content reflect perturbations in tertiary structure and disulfide bond rearrangement [4]. As shown in Figure 2, increasing sodium bicarbonate concentration significantly increased (*p* < 0.05) the active sulfhydryl content of pork myosin. However, no significant difference (*p* > 0.05) was observed between 40 mM and 50 mM supplementation, while total sulfhydryl content exhibited a progressive decline across the 30–50 mM range. This phenomenon may be attributed to the rise in pH and ionic strength of the pork myosin solution with increasing sodium bicarbonate, leading to the structural changes in myosin that expose internal sulfhydryl groups to the surface of myosin molecules, thereby increasing active sulfhydryl content [15]. Additionally, in an alkaline environment, structural modifications in myosin may result in the oxidation of some sulfhydryl groups or the formation of disulfide bonds through reactions between sulfhydryl groups, consequently reducing total sulfhydryl content [8]. Supporting this, Hunt and Park [20] demonstrated that sodium bicarbonate could effectively substitute for phosphate in mincemeat by enhancing its pH value, thereby promoting the cross-linking of myosin heavy chains. Similarly, Saleem Hasnain and Ahmad [9] further revealed that increasing sodium bicarbonate concentration induces conformational changes in chicken myosin, exposing more active sulfhydryl groups on the protein molecule surface.

### 3.5. Surface Hydrophobicity

**Figure 3:**
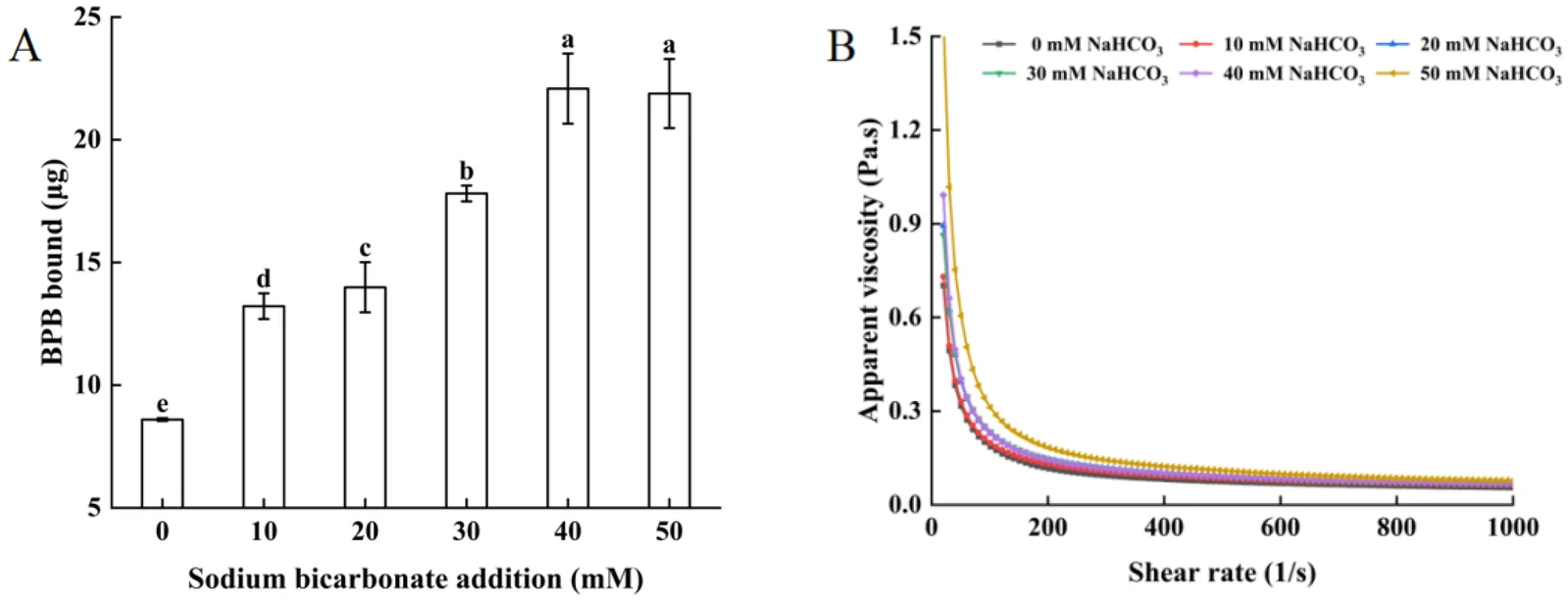
Changes in the surface hydrophobicity (**A**) and apparent viscosity (**B**) of pork myosin with different sodium bicarbonate concentrations. Each value represents the mean ± SE, *n* = 3. (a–e): Different parameter superscripts indicate significant differences (*p* < 0.05).

Surface hydrophobicity is a sensitive probe of protein conformational integrity and structural flexibility [21]. As depicted in Figure 3A, the surface hydrophobicity of pork myosin was significantly enhanced (*p* < 0.05) with increasing sodium bicarbonate. No significant difference (*p* > 0.05) was observed between supplementation levels of 40 mM and 50 mM. This result may be attributed to the dissociation of sodium bicarbonate in solution, which elevates the pH of the medium. Under alkaline conditions, certain non-covalent interactions between myosin molecules are disrupted, altering the charge distribution on the protein surface and enhancing electrostatic repulsion between molecules [22]. Consequently, myosin undergoes partial unfolding, exposing previously buried hydrophobic residues to the solvent and increasing surface hydrophobicity. Notably, Xiong et al. [23] demonstrated that adding sodium bicarbonate could modify the pH of chicken breast curing solutions, causing the isoelectric point of chicken proteins to shift. This deviation results in hydrophobic amino acid residues relocating toward the protein interior. Likewise, Zhu et al. [14] confirmed that sodium bicarbonate incorporation induces structural expansion in chicken batter proteins, enhancing hydrophobic interactions and promoting gel network formation.

### 3.6. Apparent Viscosity

The apparent viscosity serves as an indicator reflecting protein structural changes to some extent. As illustrated in Figure 3B, all samples exhibited shear-thinning behavior (i.e., decreasing apparent viscosity with increasing shear rate), consistent with typical viscoelastic protein solutions. At any given shear rate, the apparent viscosity of pork myosin increased as sodium bicarbonate concentration rose from 0 mM to 50 mM. The dissolution of sodium bicarbonate raises both the pH and ionic strength, strengthening active sulfhydryl groups and surface hydrophobicity while reducing electrostatic repulsion between them. This reducing the turbidity and particle size (Figure 1) of the myosin solution, forming a more stable protein structure and consequently enhancing protein apparent viscosity. Liu et al. [11] observed that as ionic strength increased, the viscosity coefficient of fish actomyosin solutions increased, with significantly higher values at high ionic strengths compared to low ones.

### 3.7. Ultraviolet Absorption Spectra

**Figure 4:**
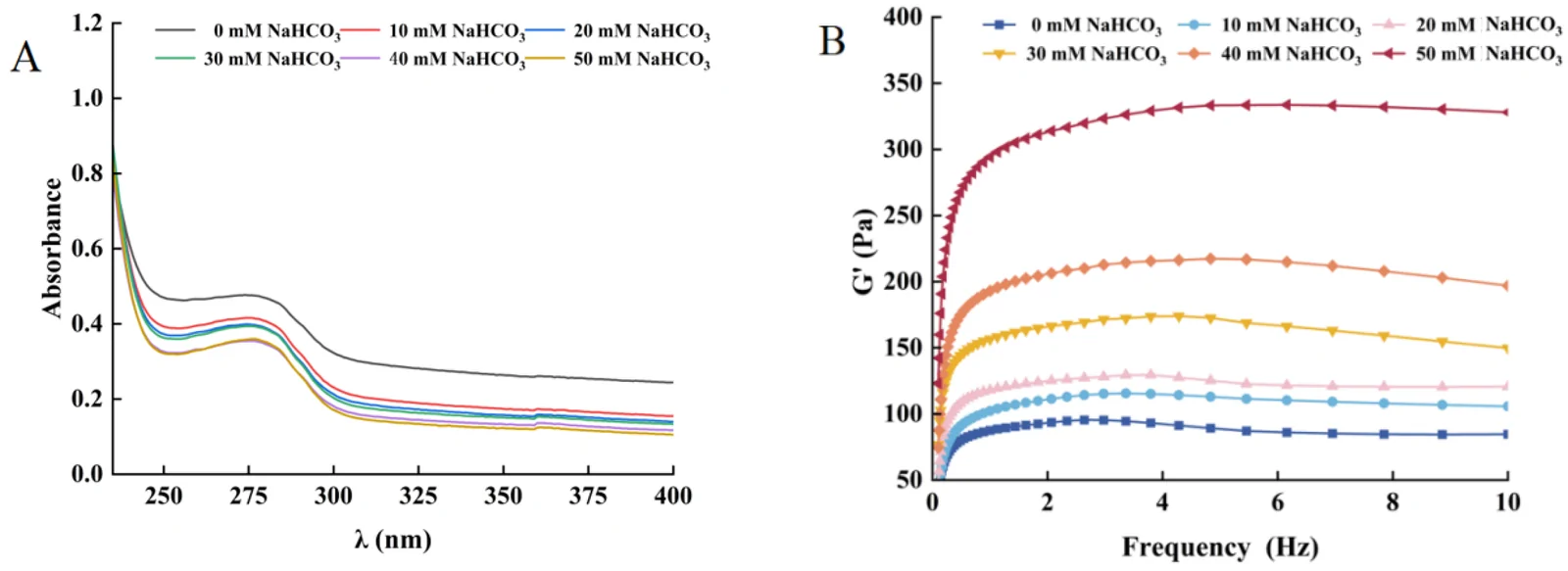
Changes in the ultraviolet spectrogram (**A**) and storage modulus (G′) (**B**) of pork myosin with different sodium bicarbonate concentrations.

Tryptophan, tyrosine, and phenylalanine residues absorb ultraviolet light predominantly near 280 nm, with tryptophan contributing most strongly to the spectral signal around 278 nm; thus, UV absorption maxima and peak intensities provide insight into the local microenvironment and solvent exposure of these aromatic residues [24]. Figure 4A shows a pronounced absorption peak centered at ~278 nm for all samples. The UV absorption spectrum peak value was minimal when sodium bicarbonate was added at concentrations of 40 mM and 50 mM, with no difference between these two conditions. This suggests that sodium bicarbonate alters the ionic strength of the solution; higher ionic strength influences the hydration layer of protein molecules, thereby modifying their conformation and affecting the microenvironment of aromatic amino acid residues, which in turn impacts their UV absorption characteristics.

### 3.8. Dynamic Rheological Properties

Rheological properties constitute a key functional parameter for characterizing protein behavior during meat processing. As shown in Figure 4B, the G′ values of myosin solutions in all samples exhibit an upward trend with increasing frequency, followed by gradual stabilization. Notably, the highest G′ value was observed at a sodium bicarbonate concentration of 50 mM. This result may arise because sodium bicarbonate elevates the pH and ionic strength of the protein solution, thereby shielding electrostatic interactions between myosin molecules and promoting the formation of hydrophobic interactions and hydrogen bonds among them. Consequently, molecular structure was expanded, improving the viscoelasticity and storage modulus of the system. Moreover, myosin exhibited weak gel-like behavior, and as frequency increased, the effective relaxation time of myosin diminished, leading to insufficient slip time between protein molecules [25].

### 3.9. Whiteness

**Figure 5:**
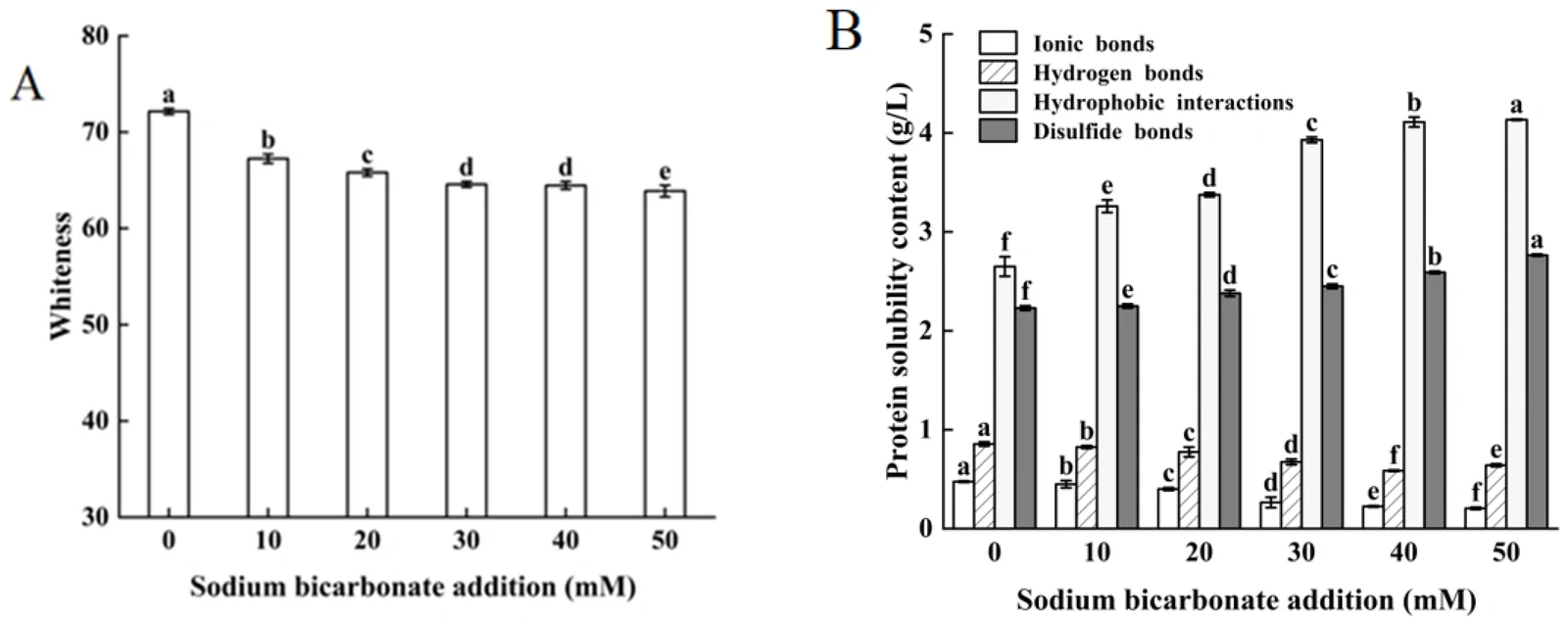
Changes in the whiteness (**A**) and chemical forces (**B**) of pork myosin gel with different sodium bicarbonate concentrations. Each value represents the mean ± SE, *n* = 3. (a–f): Different parameter superscripts indicate significant differences (*p* < 0.05).

Figure 5A presents the influence of sodium bicarbonate on the whiteness of pork myosin gels. Relative to the control, whiteness declined significantly (*p* < 0.05) with increasing sodium bicarbonate concentration. However, no significant difference (*p* > 0.05) was observed between the supplementation levels of 30 mM and 40 mM. This phenomenon may be attributed to the enhanced alkalinity of the system with increasing sodium bicarbonate, which induces changes in the molecular structure of myosin. These structural alterations affect light scattering and absorption, thereby reducing whiteness. Additionally, alkaline conditions may promote chemical reactions between myosin and other components, further influencing the network structure and optical properties of the gel, leading to a decrease in whiteness. Consistent findings have been reported in similar studies [26], indicating that as sodium bicarbonate levels increase, the L* and b* values of marinated raw beef and goat meat significantly decrease. Similarly, Chang et al. [5] demonstrated that higher pH values can slow down the thermal denaturation of myoglobin during cooking, resulting in darker meat color.

### 3.10. Chemical Forces

Gel formation is typically induced by interaction forces such as ionic bonds, hydrogen bonds, disulfide bonds, and hydrophobic interactions between protein molecules. As shown in Figure 5B, the ionic bonds, hydrogen bonds, hydrophobic interactions, and disulfide bonds of myosin gel changed significantly (*p* < 0.05) with increasing sodium bicarbonate. Hydrophobic interactions and disulfide bonds emerged as dominant contributors to the structural integrity of pork myosin gels, whereas ionic and hydrogen bonds played comparatively minor roles, primarily supporting initial chain alignment and tail winding during early-stage gelation [13,27]. The progressive decline in ionic and hydrogen bonding is consistent with sodium bicarbonate-induced increases in net surface charge on myosin, enhancing electrostatic repulsion and reducing bond stability. Concurrently, the changes in pH altered the hydration environment and molecular conformation of myosin molecules, modifying the position and activity of groups involved in hydrogen bond formation, thus reducing the number or strength of ionic and hydrogen bonds. In contrast, hydrophobic interactions and disulfide bond content increased progressively: alkaline conditions promoted partial unfolding of myosin, exposing buried hydrophobic residues and enhancing hydrophobic association [28]; simultaneously, sulfhydryl groups were more prone to oxidation, forming disulfide bonds and increasing their abundance.

## 4. Conclusions

The results demonstrated that sodium bicarbonate supplementation markedly increased the pH of pork myosin solutions and induced substantial conformational rearrangements. This structural changes was evidenced by decreased turbidity and particle size, as well as a decline in total sulfhydryl content. Concomitantly, surface hydrophobicity and active sulfhydryl content rose, reflecting enhanced exposure of hydrophobic domains and reactive thiol residues following partial unfolding. These alterations collectively strengthened hydrophobic interactions and promoted disulfide bond formation, while weakening ionic and hydrogen bonding networks of myosin gels. As a result, the apparent viscosity and G′ increased, peak UV absorbance decreased, and gel whiteness diminished. In conclusion, adding sodium bicarbonate improved molecular forces and rheological properties of pork myosin.

## Data Availability

The original contributions presented in the study are included in the article; further inquiries can be directed to the corresponding author.
